# Popliteal lymph node dissection for metastatic squamous cell carcinoma: a case report of an uncommon procedure for an uncommon presentation

**DOI:** 10.1186/1477-7819-9-130

**Published:** 2011-10-15

**Authors:** Basem B Morcos, Sameh Hashem, Firas Al-Ahmad

**Affiliations:** 1 Department of Surgical Oncology, King Hussein Cancer Center, Queen Rania Al Abdullah Street, P.O.Box 1269 Al-Jubeiha, Amman 11941, Jordan; 2 Department of Radiation Oncology, King Hussein Cancer Center, Queen Rania Al Abdullah Street, P.O.Box 1269 Al-Jubeiha, Amman 11941, Jordan

**Keywords:** Squamous cell carcinoma, Popliteal metastasis, Popliteal dissection

## Abstract

Lymph node metastasis from cutaneous squamous cell carcinoma is uncommon. The popliteal fossa is rarely involved with metastasis. Popliteal lymph node dissection is uncommonly performed and not frequently discussed in the literature. We present a case of squamous cell carcinoma of the heel with popliteal and inguinal metastasis. This is followed by a description of the relevant anatomy of the popliteal fossa and the technique of popliteal lymphadenectomy.

## Background

Regional lymph node (LN) metastasis is an important prognostic factor for skin cancers like melanomas and squamous cell carcinomas (SCC) [1]. The usual nodal basins involved are the cervical, axillary and inguinal regions. However, metastasis can involve other areas, like the epitrochlear and popliteal regions. The use of preoperative lymphoscintigrapy (LS) has allowed the detection of interval or in-transit LN, which can also be involved with metastatic disease [1].

Skin cancers of the lower limb below the knee mainly metastasise to the groin. Metastasis to the popliteal LN is much less common. In melanoma, the incidence of popliteal metastasis ranges from 0.3% to 7% [2-4]. For SCC the incidence is not known, but appears to be less than these figures. Lymph nodes in this region should be managed as those in any other nodal basin. They should be looked for, and if involved, they should be resected. Due to the fact that popliteal node metastasis is uncommon and that many surgeons lack the experience in popliteal dissection, this issue is infrequently discussed in the literature. The wider adoption of sentinel lymph node (SLN) biopsy for skin cancer is causing more surgeons to deal with LN in this region. We present a case of SCC with popliteal LN metastasis. This is followed by a review of the relevant anatomy and the surgical technique of popliteal lymphadenectomy.

## Case presentation

In 2008, a 41-year-old lady developed an ulcer on her left heel. This was excised by a general surgeon at another hospital. Pathological examination revealed a 3.8 cm, moderately differentiated SCC; the margins were involved. No further treatment was offered to the patient. A year later, in June 2009, the tumor recurred and was excised again; the margins were negative this time.

**Figure 1:**
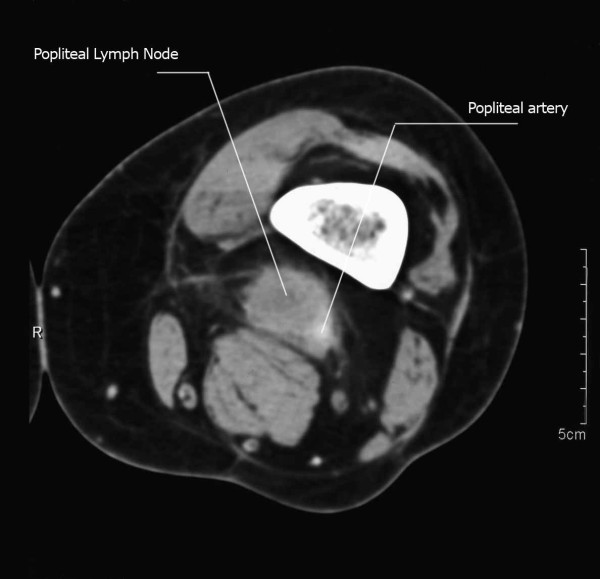
**An MRI showing the popliteal metastasis**.

Two months later she was referred to our center for further management. On examination she had a small non-healing ulcer on the left heel. There were palpable LN in the popliteal and inguinal regions of the same limb. An MRI was done for the popliteal and inguinal regions (Figure 1). It showed multiple inguinal LN and a 3-cm popliteal mass partially encasing the popliteal artery. A CT scan of the chest and liver did not reveal any distant metastasis. A biopsy was taken from the heel ulcer, and it confirmed the presence of residual SCC.

**Figure 2:**
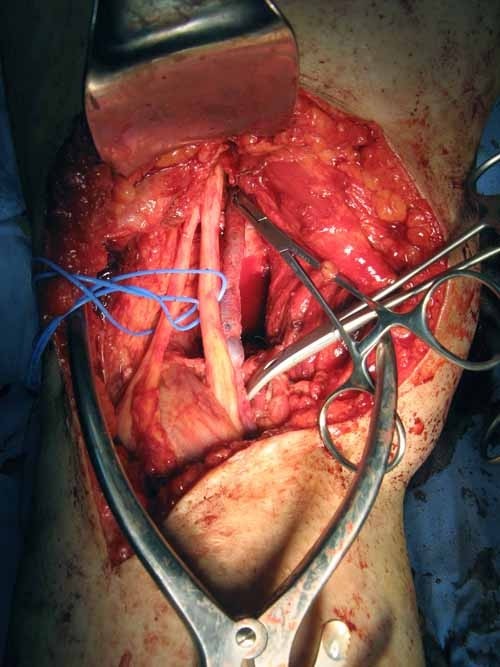
**This picture shows the dissected popliteal fossa**. The popliteal artery is excised together with the tumor mass.

**Figure 3:**
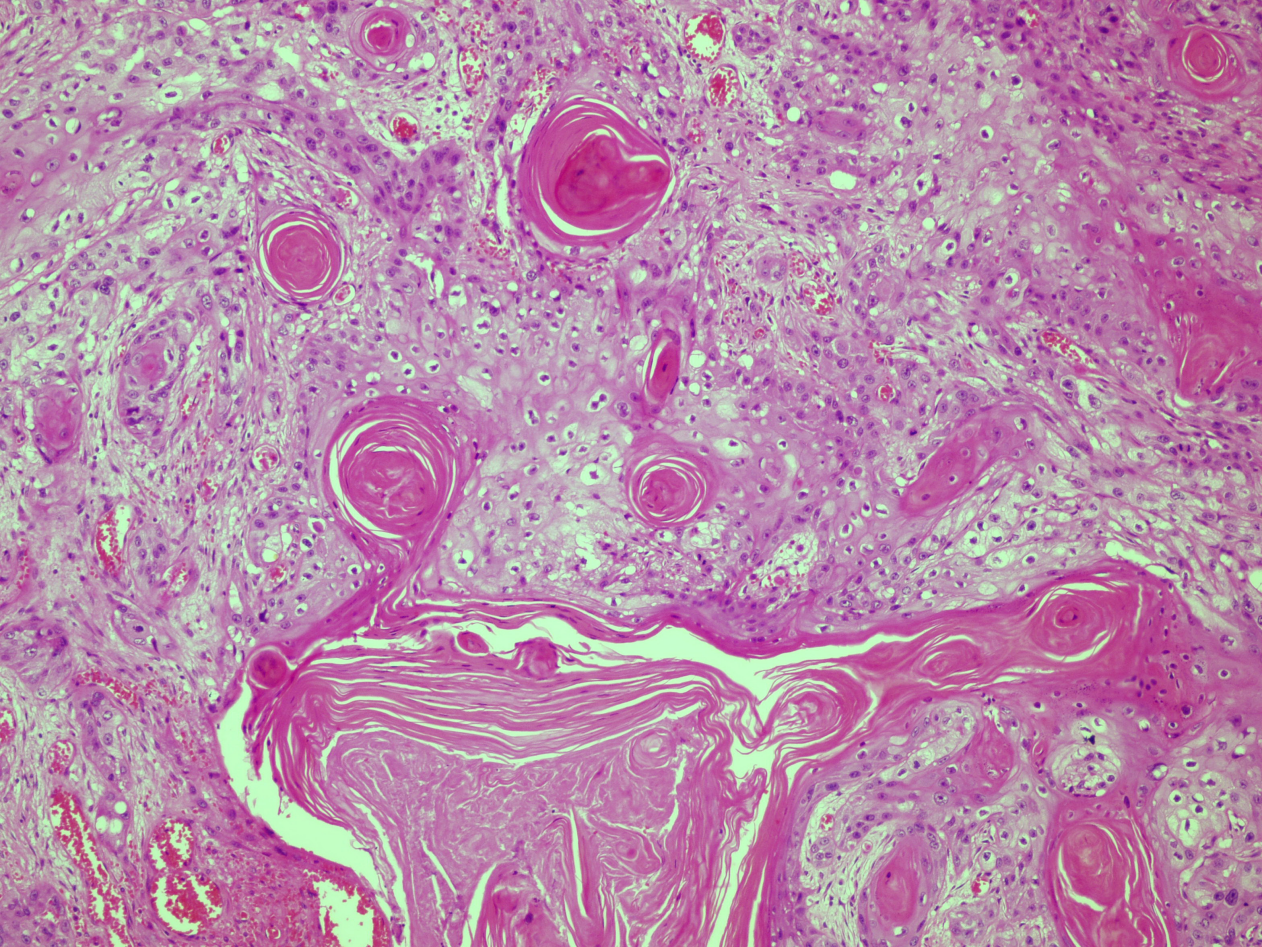
**Hematoxylin and eosin (H&E) slide showing the primary tumor (see text)**.

**Figure 4:**
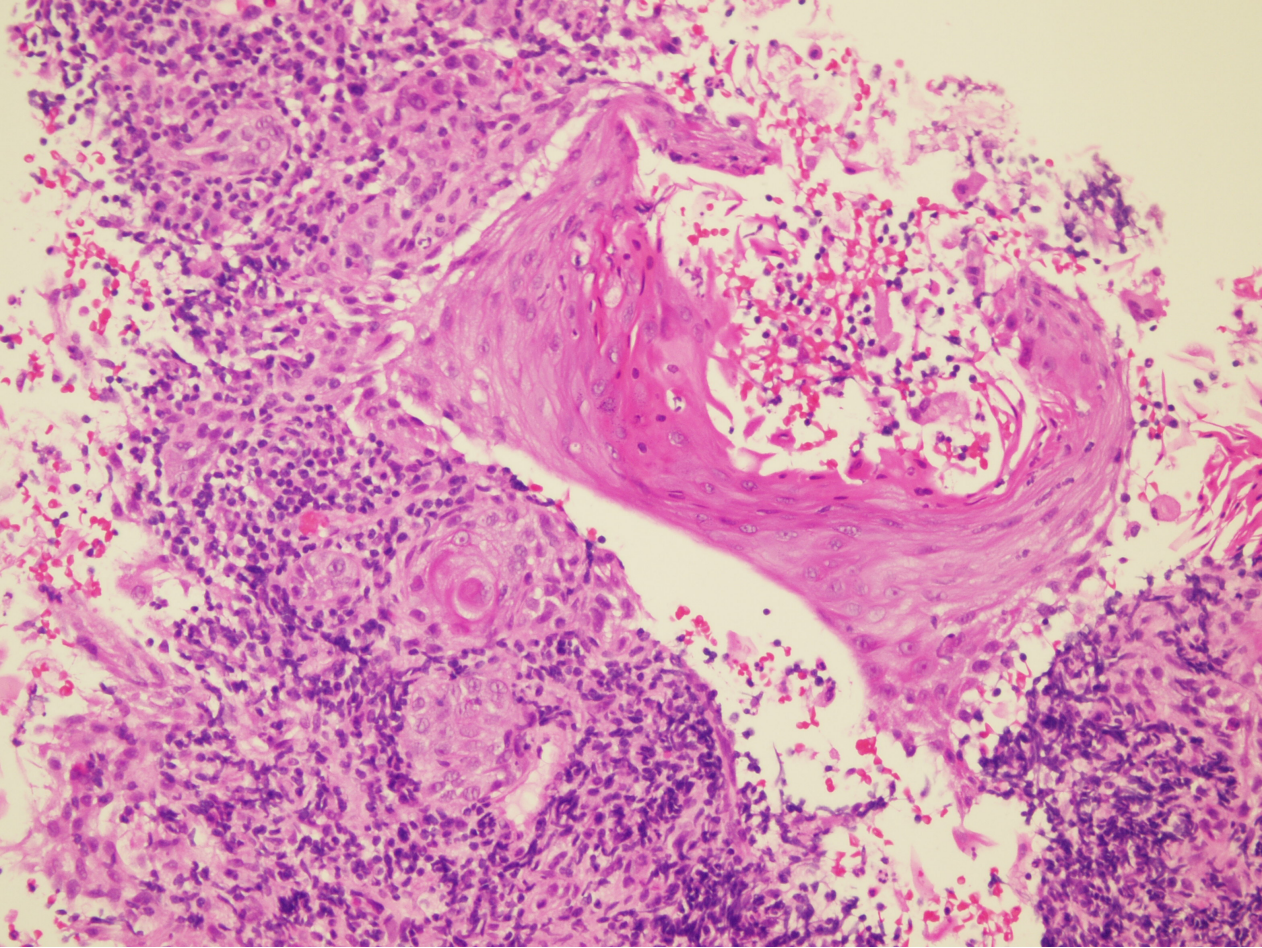
**H&E slide showing a tumor deposit in a LN (see text)**.

A wide local excision of the tumor as well as popliteal and groin dissections were performed. The ulcer was excised with a wide margin including part of the calcaneus. The resulting defect was covered with a flap. The patient underwent popliteal dissection in the prone position. The popliteal mass and LN were removed together with the adherent segment of the popliteal artery (Figure 2). A reconstruction was then undertaken with an autologous saphenous vein graft. The groin dissection was performed with the patient in supine position and it included the femoral, iliac and obturator LNs. The patient recovered well, and aside from a small seroma in the groin, she had no complications. The histopathologic examination revealed an ulcerated, moderately differentiated squamous cell carcinoma showing moderate atypia, few mitotic figures and keratin pearls (Figure 3). The tumor was completely excised with negative margins. There were 3 involved popliteal LNs, one of them completely replaced by tumor, and 13 involved inguinal LNs out of the 32 removed. The tumor in the LN showed similar morphology to the primary tumor (Figure 4). Because of the extensive lymphatic involvement, the patient was offered radiotherapy to the region. As expected, she developed significant lymphoedema, but remained ambulatory without assistance when she was last seen 11 months after the operation.

### Popliteal lymph node dissection

**Figure 5:**
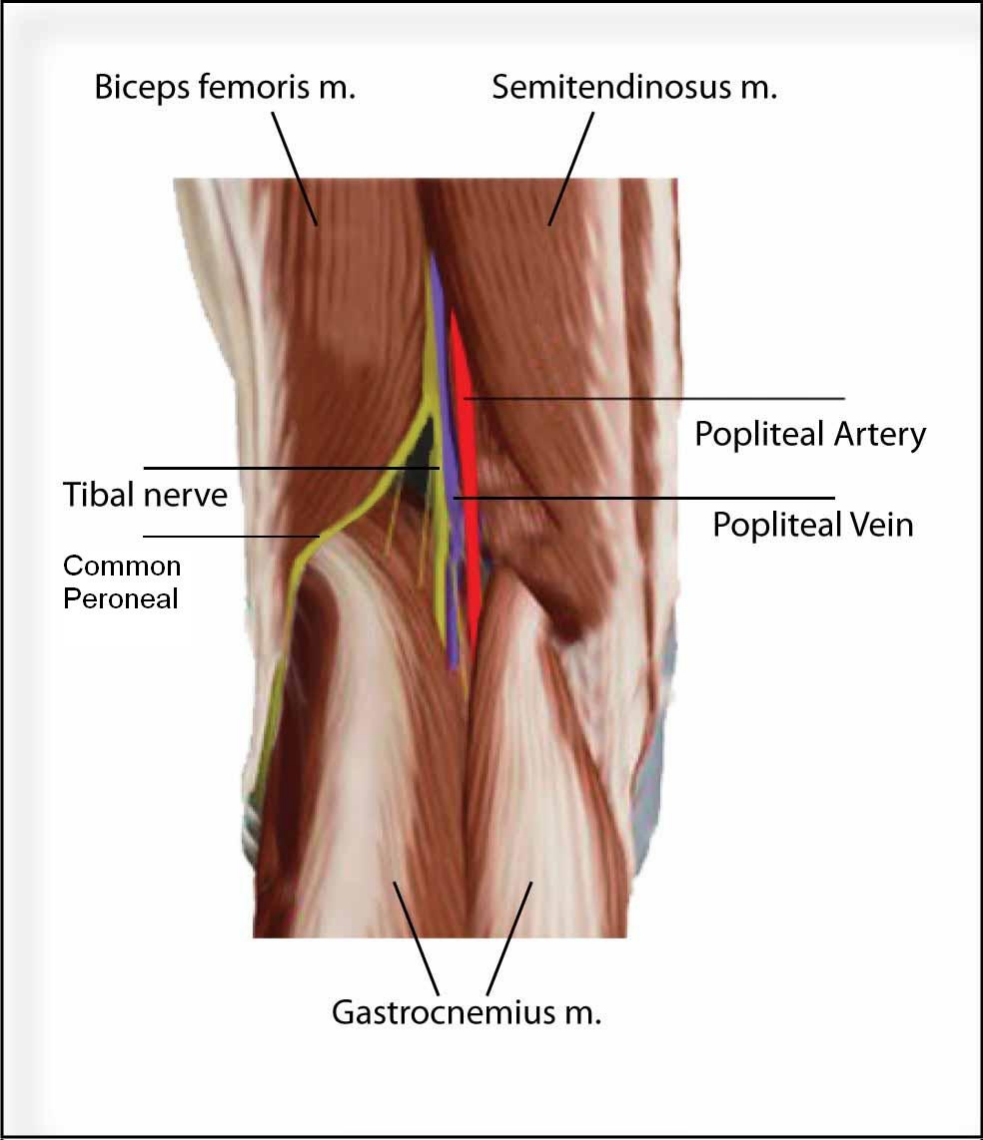
**Anatomy of the left popliteal fossa**.

The popliteal fossa is a diamond-shaped area bound superiorly by the heads of both the Biceps Femoris and Semimembranosus muscles and inferiorly by the 2 heads of Gastrocnemius muscle (Figure 5). This space is covered by the tough popliteal fascia and contains the popliteal LNs in addition to the popliteal neurovascular bundle. The neurovascular structures pass through the fossa in the middle. The LNs are contained in the fatty tissue that lies along the vessels. For adequate LN dissection, all of the fatty content should be removed. There is usually 1 LN in the subcutaneous tissue, usually in relation to the site where the small saphenous vein crosses the fascia. Making extra-fascial skin flaps ensures excision of this LN. The popliteal LNs are usually 2 to 7 in number [3-5].

In general there are 2 steps to popliteal LN dissection. The first step is adequate exposure of the diamond shaped fossa. This should include careful identification and preservation of the neurovascular structures. Once that is done, dissection of the fat pad should be performed thoroughly.

**Figure 6:**
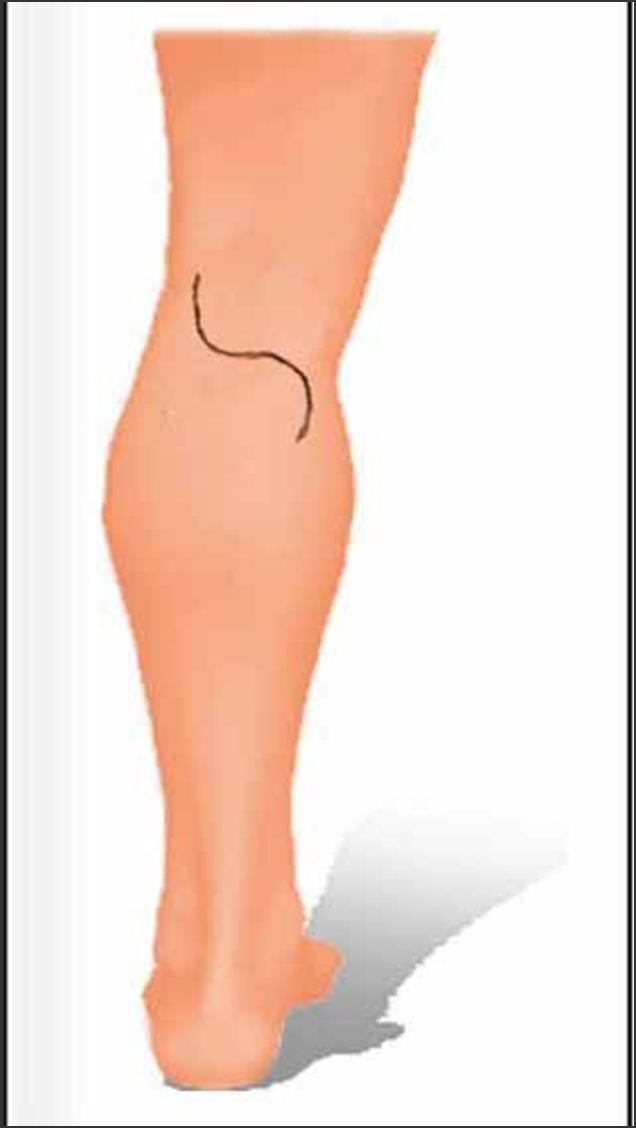
**Incision for popliteal lymph node dissection**.

The patient is positioned prone with the knees slightly flexed on a pillow. The posterior approach offers the best exposure for this procedure [6]. The skin is incised in an S-shaped fashion, with the transverse limb over the posterior knee crease (Figure 6). The cranial extension is made laterally and the caudal extension medially. It is imperative that the incision does not cross the transverse crease; doing so might lead to some contracture of the knee joint, especially if the patient is to receive postoperative radiotherapy to the region. This incision can be modified in some cases to incorporate a preceding scar of the SLN biopsy procedure; that's why the incision for a SLN biopsy should be carefully planned. Skin flaps are dissected down to the popliteal fascia. During dissection of the lower part of the flaps 2 structures are found: the small saphenous vein and the medial Sural nerve. The vein is usually ligated and divided. Preservation, however, is sometimes possible. In some cases the vein will not be encountered at this stage because it crosses the fascia at a lower level. The medial Sural nerve, which arises from the Tibial nerve, usually passes behind the vein and it should be preserved if possible. In some cases this is not possible, and this results in an area of cutaneous anesthesia on the lateral aspect of the foot and ankle.

Subsequently, the popliteal fossa is entered by opening the popliteal fascia vertically in the midline. The first structure to appear is the Tibial nerve, which crosses vertically and disappears between the 2 heads of Gastrocnemius. Lateral to it is the Peroneal nerve, which courses laterally along the biceps femoris tendon and then turns obliquely towards the fibula. Both nerves should be retracted laterally with vessel loops to allow adequate and safe exposure of the vessels and the LNs. Dissection deep to the Tibial nerve will reveal the popliteal vein. The popliteal artery passes deep and slightly medial to the vein. It is the deepest structure in the popliteal fossa and lies on the femur and capsule of the knee joint. The LNs are contained within the fatty tissue that is found superficial, alongside and deep to the popliteal vessels. LN dissection should include, therefore, all the fatty tissue in the popliteal fossa till reaching the posterior aspect of the knee joint. It should be noted that the popliteal fossa is wider in the deeper part than in the superficial part. Adequate retraction of the Gastrocnemius muscle heads, therefore, is essential to ensure adequate exposure and dissection of all the LNs.

After securing hemostasis, the fascia is approximated and the wound is closed over a negative suction drain. A knee splint or back slab is preferably applied at this stage and left in for the first few postoperative days. Postoperative Lymphoedema is expected in some patients [4]. The incidence of lymphoedema is expected to increase dramatically with the addition of inguinal dissection and or radiotherapy.

## Discussion

We describe an unusual case. The incidence of LN metastasis from SCC is relatively uncommon. It ranges from 0.5% to 16% [7], with most series reporting a rate of < 5% [8]. Usually this occurs in tumors with poor prognostic factors such as larger size, poor differentiation, presence of perineural invasion or recurrent tumors. Popliteal metastasis is quite rare. In a series of 1, 118 patients, with localized squamous cell carcinoma of the extremity, treated at M.D. Anderson Hospital and Tumor Institute in Houston, Texas, only 16 patients (1.4%) developed recurrence [9]. Of the 106 treated patients who had regional or distant metastasis, only 2 patients had popliteal metastasis, and both had inguinal metastasis as well.

Although SLN biopsy is mainly indicated for melanomas, it is gaining wider application in the management of other skin tumors, like SCC [10]. Still, the indications for SLN biopsy in SCC are not clear. Its' use in lower extremity tumors will allow the detection of more patients with popliteal LN metastasis, many of those will probably be micrometastases.

The procedure of popliteal lymphadenectomy was first described by Karakousis in 1980. Most later descriptions are based on it. Knowledge of the relevant anatomy and the surgical technique of popliteal LN dissection is important to avoid injury to important structures and to allow adequate LN dissection.

## Conclusion

Popliteal metastasis from skin SCC is quite uncommon. It usually occurs in patients with advanced or recurrent tumors. Appropriate management is not clear, but surgical dissection, in the absence of distant metastasis, is indicated since there is no known effective adjuvant treatment for skin SCC.

## Consent

Written informed consent was obtained from the patient for publication of this Case report and any accompanying images. A copy of the written consent is available for review by the Editor-in-Chief of this journal.

## Competing interests

The authors declare that they have no competing interests.

## Authors' contributions

FA has reviewed and written part of the case report. SH arranged and provided the artwork and picture. BM conceived of the study and wrote the majority of the paper. All were involved in the design and review of the paper and all read and approved the final manuscript.
